# A Single-Nucleotide Polymorphism in Serine-Threonine Kinase 11, the Gene Encoding Liver Kinase B1, Is a Risk Factor for Multiple Sclerosis

**DOI:** 10.1177/1759091415568914

**Published:** 2015-02-17

**Authors:** Anne I. Boullerne, Demetrios Skias, Elizabeth M. Hartman, Fernando D. Testai, Sergey Kalinin, Paul E. Polak, Douglas L. Feinstein

**Affiliations:** 1 Department of Anesthesiology, University of Illinois at Chicago, IL, USA; 2 Department of Veterans Affairs, Jesse Brown VA Medical Center, Chicago, IL, USA; 3 Department of Neurology, University of Illinois at Chicago, IL, USA; 4 Center for Neurosciences, Orthopedics and Spine, Dakota Dunes, SD, USA

**Keywords:** multiple sclerosis, liver kinase B1, single-nucleotide polymorphism

## Abstract

We identified a family in which five siblings were diagnosed with multiple sclerosis (MS) or clinically isolated syndrome. Several women in the maternal lineage have comorbidities typically associated with Peutz Jeghers Syndrome, a rare autosomal-dominant disease caused by mutations in the serine-threonine-kinase 11 (STK11) gene, which encodes liver kinase B1. Sequence analysis of DNA from one sibling identified a single-nucleotide polymorphism (SNP) within STK11 intron 5. This SNP (dbSNP ID: rs9282860) was identified by TaqMan polymerase chain reaction (PCR) assays in DNA samples available from two other siblings. Further screening was carried out in samples from 654 relapsing-remitting MS patients, 100 primary progressive MS patients, and 661 controls. The STK11-SNP has increased frequency in all female patients versus controls (odds ratio = 1.66, 95% CI = 1.05, 2.64, *p* = .032). The STK11-SNP was not associated with disease duration or onset; however, it was significantly associated with reduced severity (assessed by MS severity scores), with the lowest scores in patients who also harbored the HLA-DRB1*1501 allele. *In vitro* studies showed that peripheral blood mononuclear cells from members of the family were more sensitive to the mitochondrial inhibitor metformin than cells from MS patients with the major STK11 allele. The increased association of SNP rs9282860 in women with MS defines this variant as a genetic risk factor. The lower disease severity observed in the context of HLA-DRB1*1501 combined with limited *in vitro* studies raises the provocative possibility that cells harboring the STK11-SNP could be targeted by drugs which increase metabolic stress.

## Introduction

Genetic factors are known to influence the risk of developing multiple sclerosis (MS), based on the increased risk in relatives of MS patients compared to the general population, and with a concordance rate in monozygotic twins around 18% in Northern latitudes above 41°, and up to 25% in families with Celtic or Scandinavian descent (Islam et al., 2006; O’Gorman et al., 2013). Early linkage studies in extended families identified the HLA locus as a risk factor (Jersild et al., 1972), which was subsequently confirmed by the first genome-wide association studies (GWAS) in Caucasians (Hafler et al., 2007). Several studies have identified the HLA-DRB1*1501 allele as the variant associated with increased risk, having an odds ratio (*OR*) of approximately 3 across various ethnic groups including Caucasian, Japanese, Middle Eastern, and African descent (Hafler et al., 2007; Schmidt et al., 2007). The rarity of families with multigeneration cases of MS, or with four or more affected members within the same family has limited further linkage analyses (Dyment et al., 2002). Instead, large cohort GWASs have identified more than 100 additional genetic risk factors (Sawcer et al., 2011; Beecham et al., 2013), many of which are implicated in immune responses and lymphocyte physiology. However, it is estimated that these factors together cannot account for more than 25% of familial inheritance, leaving much of the inheritability of MS to be identified.

During the course of recruiting MS patients, we identified a family (MSF) in which five children, but neither parent, had been diagnosed with MS or clinically isolated syndrome (CIS), or suspected of having MS. Evaluation of two members of the family ascertained their diagnosis of MS; and blood samples were made available from three of the five siblings. Interestingly, comorbidities in the maternal lineage include breast cancer, colon cancer, ovarian cancer, and colon polyps, symptoms associated with Peutz Jegher Syndrome (PJS)—a rare autosomal-dominant disease characterized by gastrointestinal polyps, mucocutaneous spots, and increased risk for cancers at several sites including breast, colon, and ovary (Launonen, 2005). The gene responsible for PJS is STK11 (serine-threonine-kinase 11) (Hemminki et al., 1998; Katajisto et al., 2007), which codes for liver kinase B1 (LKB1), a tumor suppressor gene (Fan et al., 2009). LKB1 functions upstream of at least 14 other protein kinases and, in that sense, is considered a master kinase with roles in numerous cellular functions (Boudeau et al., 2003). LKB1 plays important roles in Tcell biology, as demonstrated by studies showing that lack of LKB1 in thymocytes reduces proliferation and induces apoptosis, and that surviving thymocytes have increased Tcell activation and increased production of Th1/Th17 cytokines (Mciver et al., 2011). LKB1 also regulates activation of adenosine monophosphate kinase (AMPK), essential to help maintain metabolic homeostasis during periods of increased cellular activities and reduced ATP levels (Alexander and Walker, 2011; Sebbagh et al., 2011), as occurs in rapidly dividing tumor cells (Han et al., 2013) as well as in activated lymphocytes (Shaw et al., 2004). AMPK can regulate activation of the mTOR complex, which in addition to its well-characterized role in protein synthesis also has roles in oligodendrocyte maturation and myelin formation (Tyler et al., 2009; Dai et al., 2014). Finally, AMPK also regulates inflammatory responses in glial cells (Dello-Russo et al., 2013), and AMPK inhibition ameliorates disease symptoms in experimental autoimmune encephalomyelitis, an animal model of MS (Paintlia et al., 2006). The wide array of functions regulated by LKB1 that have been implicated in the pathogenesis of MS suggest that alterations in LKB1 expression or activity may be a contributing factor to MS disease.

While a direct role for LKB1 in MS has not yet been described, in one study (Sun et al., 2011), conditional knockout of LKB1 from a subset of spinal cord neurons produced mice with MS type symptoms including demyelination and macrophage infiltration. More recently, conditional knockout of LKB1 from Schwann cells was found to be essential for myelination of peripheral axons (Pooya et al., 2014; Shen et al., 2014). The mechanisms of action responsible for reduced peripheral myelination are not fully known, but were postulated to be due to metabolic impairment and reduced levels of citrate, necessary for lipid production (Pooya et al., 2014), or to disruption of Schwann cell: axon interfaces which involve binding of LKB1 to the polarity protein Par-3 (Shen et al., 2014).

In the current article, we tested the possibility that members of the MSF harbored a polymorphism in the STK11 gene that could on the one hand help account for comorbidities and at the same time represent a genetic risk factor for increased risk of MS. DNA sequence analysis of available samples allowed us to identify a single-nucleotide polymorphism (SNP) located in intron 5 of the STK11 gene (STK11-SNP). The SNP (dbSNP ID: rs9282860) was confirmed by PCR analysis and then used to determine its prevalence in DNA samples from 654 relapsing-remitting MS patients, 100 primary progressive MS patients, and 661 controls. Our results show that the STK11 intron 5 SNP is a risk factor for MS in women with an *OR* of 1.66. Furthermore, while the presence of the STK11-SNP did not influence neurological severity (as assessed by the MS severity score, MSSS), the average MSSS values were lower in patients who harbored both the STK11-SNP and the HLA-DRB1*1501 risk allele.

## Material and Methods

### Subjects and DNA Samples

Informed consent was obtained from all MS patients seen at the Department of Neurology at University of Illinois at Chicago (UIC). Three patients of the identified MS family from Hispanic descent (MSF1, MSF2, and MSF4) and an additional two remitting-relapsing MS (RRMS) patients and one control from European descent were enrolled. Clinically definite MS was diagnosed according to the revised Mc Donald criteria (Polman et al., 2011). Blood samples were collected, DNA isolated, and used for sequence analysis and TaqMan PCR assays. All procedures were approved by the UIC Institutional Review Board.

DNA samples from 650 RRMS patients, 100 primary progressive MS (PPMS), patients and 650 controls, all of European descent, were generously provided by Dr. Jorge Oksenberg (Department of Neurology, University of California, San Francisco). Information for these cohorts included clinical parameters of MSSS (Roxburgh et al., 2005), age at onset of first episode of neurological dysfunction suggestive of demyelinating disease, disease duration from onset, self-reported comorbidities for the patient and first-degree relatives, and the presence or absence of the HLA-DRB1*1501 allele (Caillier et al., 2008). An additional 12 DNA samples (2 RRMS and 10 controls) were isolated from tissue samples obtained from the Human Brain and Spinal Fluid Resource Center (VA Greater Los Angeles, CA).

### DNA Sequencing

DNA was isolated from 350 µL of whole blood using Purelink gDNA Blood Kit according to the manufacturer’s instructions (Life Technologies, Grand Island, NY). Purified genomic DNA was used as template for PCR amplification of the STK11 exonic and proximal intronic regions which were then sequenced by capillary electrophoresis. Briefly, primers were designed from the reference sequence using Primer-BLAST (Ye et al., 2012) and validated using the OligoAnalyzer (Owczarzy et al., 2008). PCR amplification was performed using the 2× AccuPrime SuperMix II (Life Technologies, Gaithersburg, MD), purified using AMPure XP beads (Agencourt Ampure, Beckman Coulter, Inc., Fullerton, CA), and then capillary electrophoresis sequencing was performed on a 96-capillary Life Technologies 3730XL DNA Analyzer Sequencer using BigDye® Terminator v3.1 Cycle Sequencing (Life Technologies, Grand Island, NY) and Mag-Bind SeqDTR (Omega Biotek, Norcross, GA). Sequence data were processed using the software package CLC genomics workbench (CLC bio, Cambridge, MA), and variants identified by visual inspection.

### TaqMan Analysis for the STK11 IV-5 SNP

A TaqMan quantitative PCR assay was used to identify the STK-11 SNP C/T variants. Genotyping was performed using a TaqMan assay targeting the identified SNP rs9282860 (Life Technologies assay C_25599132_10). PCRs were performed in 20 µL reactions on an Applied Biosystems ViiA7 instrument, using the TaqMan Genotyping Master Mix according to manufacturer’s instructions. Data analysis, including SNP calling, was performed using the ViiA7 software as well as Genotyper Software (Life Technologies) and verified by visual inspection. A subset of samples with either allele was validated by PCR amplification and capillary electrophoresis sequencing as above.

### Metformin Experiments

Peripheral blood mononuclear cells (PBMCs) were acutely isolated with Ficoll-Paque (GE Healthcare, Uppsala, Sweden) on Leucosep™ frit tubes (Greiner Bio-One, Monroe, NC) using standard protocols. PBMCs were plated at 250,000 cells/100 µL per well of CytoOne 96-well tissue culture plate (USA Scientific, Ocala, FL) and cultured in RPMI media containing 1% fetal bovine serum (Gibco, Grand Island, NY) with antibiotics and antimycotics (Corning, Manassas, VA). Cells were stimulated overnight with 0 or 1 µg/mL anti-CD3 and anti-CD28 antibodies (Antigenix America, Huntington Station, NY). The next day metformin (Fluka, Laramie, WY) was added at varying doses, and one day later, cytotoxicity was assessed by LDH release (Promega, Madison, WI).

### Statistical Analyses

Associations between STK11-SNP frequency and MS incidence or comorbidities were tested for by binary logistic regression, stratified by gender or HLA-DRB1*1501 allele. Associations between STK11-SNP and MS severity, onset, and duration, stratified by gender and HLA-DRB1*1501 allele were tested for by linear regression. Effects of metformin on PBMC cells were tested by one-way nonparametric analysis of variance. Comparisons in Table 1 were done using two-tailed *t* test. Comparisons in Table 3 for presence of STK11-SNP were made by Fisher’s exact test. Analyses were carried out using SPSS version 22. Statistical comparisons between MS cohorts and controls excluded data from MSF1, MSF2, and MSF4, as they are not of European descent.

**Table 1.** Case and Control Demographics.

	Total	Age (*SE*)	DD (*SE*, *n*)	Onset (*SE*, *n*)	MSSS (*SE*, *n*)
Control					
Female	425	42.5 (0.56)*			
Male	236	44.5 (0.86)			
Female:Male	1.80				
All controls	661				
RRMS					
Female	445	44.9 (0.55)	12.2 (0.50, 437)	32.6 (0.43, 437)	3.40 (0.11, 435)
Male	209	46.0 (0.90)	13.1 (0.80, 208)	33.0 (0.68, 208)	3.59 (0.17, 208)
Female:Male	2.13				
All RRMS	654				
PPMS					
Female	64	63.5 (1.12)	22.1 (1.30, 64)	41.3 (1.5, 64)	7.02 (0.12, 64)
Male	36	64.9 (1.63)	23.3 (1.96, 36)	41.6 (2.0, 36)	6.76 (0.17, 36)
Female:Male	1.78				
All PPMS	100				

*Note.* Age = mean age at enrollment in years; DD = mean disease duration in years; Onset = mean age at disease onset; MSSS = mean multiple sclerosis severity score; RRMS = remitting-relapsing multiple sclerosis; PPMS = primary progressive multiple sclerosis.

* *p* < .05 vs. male controls by *t* test.

**Table 2.** Prevalence of STK11-SNP in MS Patients Versus Controls.

		STK11-SNP allele						
		C major/ancestral		T minor				
Gender	Group	N	%	N	%	Total	OR	p
Male	Control	217	91.9	19	8.1	236		
	RRMS	190	90.9	19	9.1	209	1.14	0.695
	PPMS	36	100.0	0	0.0	36	nd	nd
	All MS	226	92.2	19	7.8	245	0.96	0.904
Female	Control	395	92.9	30	7.1	425		
	RRMS	396	89.0	49	11.0	445	1.63	0.044
	PPMS	56	87.5	8	12.5	64	1.88	0.135
	All MS	452	88.8	57	11.2	509	1.66	0.032
Both	Control	612	92.6	49	7.4	661		
	RRMS	586	89.6	68	10.4	654	1.45	0.058
	PPMS	92	92.0	8	8.0	100	1.09	0.835
	All MS	678	89.9	76	10.1	754	1.40	0.079

*Note.* C = major allele; T = minor allele; nd = not determined; *OR* = odds ratio; RRMS = remitting-relapsing multiple sclerosis; PPMS = primary progressive multiple sclerosis; MS = multiple sclerosis; *p* value (binary logistic regression).

**Table 3.** Association of STK11-SNP With Tumor or Cyst-Related Comorbidities.

		STK11		Self		1st-degree relatives		1st-degree relatives	
Group	Gender	allele	Total	w/TCCP	%	w/TCCP	%	w/MS	%
RRMS	M	C T	190 19	4 0	2.1 0.0	25 5	13.2 26.3	8 2	4.2 10.5
	F	C T	396 49	18 2	4.5 4.1	49 6	12.4 12.2	7 1	1.8 2.0
	M/F	C T	586 68	22 2	3.8 2.9	74 11	12.6 16.2	15 3	2.6 4.4
	M/F	C/T	654	24	3.7	85	13.0	18	2.8
PPMS	M	C T	33 3	2 0	6.1 0.0	1 0	3.0 0.0	1 0	3.0 0.0
	F	C T	59 5	4 0	6.8 0.0	5 0	8.5 0.0	4 1	6.8 20.0
	M/F	C T	92 8	6 0	6.5 0.0	6 0	6.5 0.0	5 1	5.4 12.5
	M/F	C/T	100	6	6.0	6	6.0	6	6.0
Controls	M	C T	217 19	2 0	0.9 0.0	26 1	12.0 5.3	0 0	0.0 0.0
	F	C T	395 30	4 3	1.0 10.0 ^a^	81 7	20.5 23.3	0 0	0.0 0.0
	M/F	C T	612 49	6 3	1.0 6.1 ^b^	107 8	17.5 16.3	0 0	0.0 0.0
	M/F	C/T	661	9	1.4 ^c^	115	17.4	0	0.0

*Note.* TCCP = tumor-cancer-cyst-polyp; RRMS = remitting-relapsing multiple sclerosis; PPMS = primary progressive multiple sclerosis; MS = multiple sclerosis.

a *p* < .01 vs. control females with C allele. ^b^ *p* < .01 vs. control M/F with C allele. ^c^ *p* < .01 vs. all RRMS; Fisher’s exact test.

## Results

### Identification of a Family With High Incidence of MS

**Figure 1.**
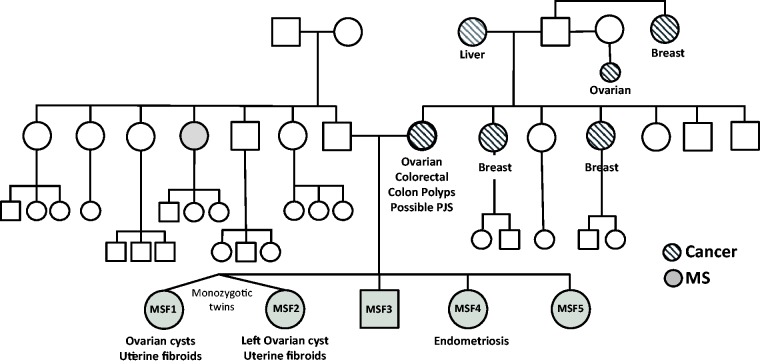
Genealogical chart of an MS family (MSF). Shaded symbols indicate family members diagnosed with MS. Hatch-filled symbols indicate family members diagnosed with cancer. Incidences of ovarian cysts, fibroids, and endometriosis, and colonic polyps are indicated. MSF1 and MSF2 are monozygotic twins. MS = multiple sclerosis.

We identified a family (MSF) in which five siblings (four females including monozygotic twins and one male) have previously been diagnosed with MS or CIS, or suspected of having MS between the ages of 23 and 26 (Figure 1). A diagnosis of RRMS in patient MSF4 was established by neurological examinations, history of relapses, and examination of MRI images which showed the presence of lesions having a typical periventricular pattern. Follow-up examination of patient MSF1 confirmed a diagnosis of RRMS on clinical grounds, and the presence of oligoclonal bands. The maternal family history is significant for malignancies of breast, ovary, and colon, but no neurological disorders in six maternal siblings, their offspring, or parents. The mother was treated for ovarian and colorectal cancers and removal of colonic polyps. Three of the four female siblings were diagnosed and treated for ovarian cysts, uterine fibroids, or endometriosis. These comorbidities are associated with PJS, a rare condition due to autosomal-dominant mutations in the STK11 gene which encodes LKB1.

### Identification of an SNP in the STK11 Gene in Members of the MSF

**Figure 2.**
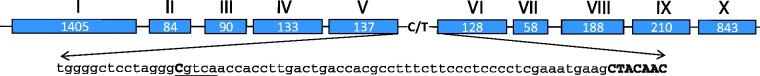
Schematic of the STK11 gene intron/exon structure. The STK11 gene consists of 10 exons. The 65 bases located at the 3′ end of STK11 intron 5 (495 bases total) are shown. The STK11-SNP is located 51 bases upstream of STK11 exon 6 (capitalized letters). The C changed to a T in the SNP is capitalized and bolded, and a consensus CRE half site is underlined.

Sequence analysis of the STK11 gene in patient MSF4 identified a SNP present at heterozygosity in intervening sequence 5 (IV5). The SNP (rs9282860, referred to herein as STK11-SNP) changes the major ancestral C allele to the minor T allele and is located 51 bp upstream from the beginning of exon 6. This area is within a regulatory region which contains potential binding sites for several transcription factors including a half site CRE motif (Figure 2). A TaqMan quantitative PCR assay confirmed the presence of this SNP in MSF4 as well from two other siblings from which samples were available (MSF1 and MSF2).

### STK11-SNP Prevalence in MS Patients

TaqMan analysis for the STK11-SNP was carried out in samples from 654 RRMS patients, 100 PPMS patients, and 661 controls. The demographics of these three cohorts are shown in Table 1. The female to male ratio was similar in all three groups (controls, 1.80; RRMS, 2.13; PPMS, 1.78). There was no significant difference between males and females for mean age at enrollment in each of the two MS groups, while control females were slightly younger than control males; or between males and females in the two MS cohorts for mean disease duration, mean age at onset, and mean MSSS. The mean age was similar between the control and the RRMS cohorts, while PPMS patients were approximately 20 years older. In agreement with the natural history of MS (Antel et al., 2012), disease onset in the PPMS group was 10 years later than in the RRMS patients.

The presence of the STK11-SNP was increased in RRMS patients versus controls (10.4% vs. 7.4%, *OR* = 1.45); however, that difference did not reach statistical significance (Table 2). Stratification by gender showed that the STK11-SNP prevalence was significantly increased in female RRMS patients compared to female controls (11.0% vs. 7.1%, *OR* = 1.63), but not in male RRMS patients. The STK11-SNP prevalence was also higher in female PPMS patients than female controls (12.5% vs. 7.1%, *OR* = 1.88); however, this increase did not reach statistical significance. In contrast to males in the control and RRMS cohorts, the STK11-SNP was not detected in any of the 36 male PPMS patients (Fisher’s test = 0.08 vs. all other males). When compared to all females with MS (RRMS and PPMS), the STK11-SNP was significantly increased (11.2% vs. 7.1%, *OR* = 1.66). These results demonstrate that the STK11-SNP is a risk factor for female patients across both RRMS and PPMS forms.

### Association of STK11-SNP With Tumor-Related Comorbidities

Because STK11-SNP was initially identified in members of the MSF who had tumor or cyst-related comorbidities, it was possible that this SNP is associated with those comorbidities, and not with MS. To test this, we examined the occurrences of cancers and cyst-related comorbidities in the three cohorts (Table 3). In RRMS patients, the STK11-SNP did not increase the percentage who had comorbidities for tumors, cancers, cysts, or polyps (3.8% in those with the C allele versus 2.9% in patients with the T allele). There was also no significant change in the percentage of first-degree relatives who had any of these comorbidities (12.6% in the C allele group vs. 16.2% in the T allele group) or who had MS. Stratification by gender suggests possible effects of STK11-SNP in males; however, those changes did not reach significance. Results for PPMS patients show that STK11-SNP reduced comorbidities; however, none of those reductions reached statistical significance. Compared to the RRMS cohort, the overall incidence of comorbidities was significantly lower in controls (3.7% vs. 1.4%). Interestingly, in controls there was a significant association of tumor and cyst-related comorbidities with STK11-SNP having an *OR* of 6.59 (P = 0.009).

### Association of STK11-SNP With HLA DRB1*1501

The highest known genetic risk factor for MS is the HLA DRB1*1501 allele, with an *OR* close to 3. We tested the hypothesis that the presence of the STK11-SNP was associated with the presence of HLA DRB1*1501 (Table 4). In the control group, HLA DRB1*1501 was present in approximately 20% of the samples, with an equal distribution in males and females; while its presence was about 2.5-fold higher in MS patients, with a slightly higher frequency in females. The STK11-SNP was present more often in HLA DRB1*1501 negative (8.0%) than in HLA DRB1*1501 positive (5.5%) controls (Fisher’s test = .001), and present equally in HLA DRB1*1501 negative and positive RRMS patients. In PPMS patients, the STK11-SNP was only found in females that were HLA DRB1*1501 negative.

**Table 4.** Association of STK11-SNP With HLA-DRB1*1501.

		HLA-DRB1*1501 negative			HLA-DRB1*1501 positive			
		STK11-SNP allele			STK11-SNP allele		
Group	Gender	C (*n*, %)	T (*n*, %)	Total	C (*n*, %)	T (*n*, %)	Total	% HLA positive
Control	Male	167 (91)	17 (9.2)*	184	42 (96)	2 (4.5)	44	19.3
	Female	313 (93)	25 (7.4)	338	78 (94)	5 (6.0)	83	19.7
	Both	480 (92)	42 (8.0)*	522	120 (95)	7 (5.5)	127	19.6
RRMS	Male	100 (92)	9 (8.3)	109	90 (90)	10 (10.0)	100	47.8
	Female	180 (89)	23 (11.3)	203	212 (90)	25 (10.5)	237	53.9
	Both	280 (90)	32 (10.3)	312	302 (90)	35 (10.4)	337	51.9
PPMS	Male	19 (100)	0 (0)	19	17 (100)	0 (0)	17	47.2
	Female	22 (73)	8 (27)**^#^**	30	34 (100)	0 (0)	34	53.1
	Both	41 (84)	8 (16)	49	51 (100)	0 (0)	51	51.0

*p = .001 vs. HLA-DRB1*1501 positive: STK11-SNP T allele, Fisher’s exact test. *^#^p* = .002 vs. female controls: STK11-SNP T allele, Fisher’s exact test.

### Influence of STK11-SNP on Disease Onset and Severity

**Figure 3.**
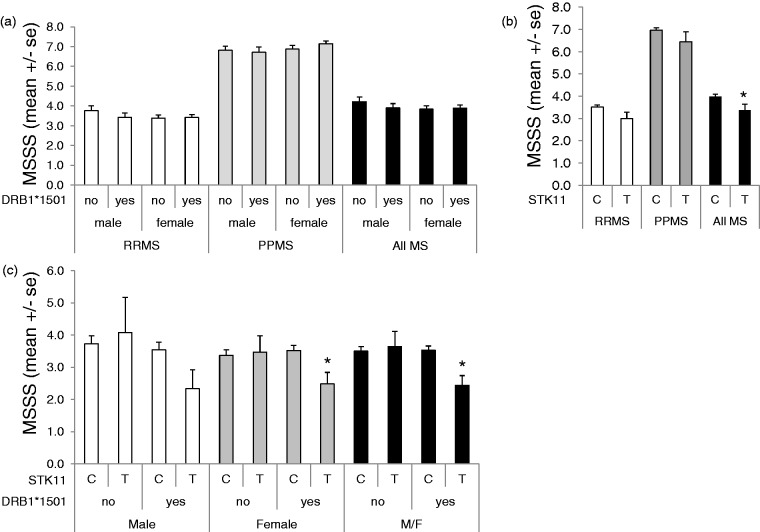
Effect of MS type, gender, HLA DRB1*1501 status, and STK11-SNP on MSSS. (a) The MSSS values for RRMS (open bars), PPMS (gray bars), and all MS (black bars) patients combined are shown stratified by gender and by HLA DRB1*1501 status. (b) The MSSS values for all RRMS patients (open bars), all PPMS patients (gray bars), and all MS (black bars) are shown stratified by STK11-SNP allele (major variant C; minor variant T). (c) MSSS values for RRMS patients, stratified by gender, HLA DRB1*1501 status (yes or no), and STK11 allele (major variant C; minor variant T). Data are mean ± *SEM*, *p < .05 vs. the C allele.

We tested if either the STK11-SNP or HLA DRB1*1501 allele alone, or together was a predictor of MSSS. Across all MS patients (RRMS and PPMS), neither gender nor the presence of HLA DRB1*1501 was a predictor of MSSS (Figure 3(a)). In contrast, the STK11-SNP was significantly associated with reduced MSSS (3.99 ± 0.10 vs. 3.37 ± 0.28, *p* = .044) across all MS patients (Figure 3(b)). In the RRMS group (Figure 3(c)), lower MSSS was associated with patients who harbored both the STK11-SNP and the HLA DRB1*1501 allele, and the association was significant in females (*p* < .05) but did not in males. A similar analysis was not carried out for PPMS patients, as there were none who harbored both the STK11-SNP and the DRB1*1501 risk allele.

Disease duration was less in male RRMS patients who expressed the STK11-SNP regardless of HLA-DRB1*1501 status; however, those reductions did not reach statistical significance (Table 5), suggesting that the relationship of STK11-SNP with MSSS (which factors in disease duration) is driven by disease severity. Disease onset was earlier in male RRMS patients who harbored the STK11-SNP and in female PPMS patients without the HLA-DRB1*1501 allele. However, neither of those reductions reached statistical significance. In both male and female RRMS, the earliest onset was in patients who harbored both the STK11-SNP and HLA DRB1*1501 allele.

**Table 5.** Association of STK11 SNP to Disease Duration and Onset.

			STK	Disease duration	Onset	
MS	Gender	HLA	allele	Mean (SEM)	Mean (SEM)	N
RRMS	M	No	C	12.61 (1.17)	33.86 (1.04)	99
			T	10.44 (4.46)	31.22 (3.72)	9
		Yes	C	14.13 (1.21)	32.78 (0.94)	90
			T	10.70^c^ (2.02)	27.60^a^ (3.10)	10
	F	No	C	12.99 (0.84)	32.39 (0.70)	177
			T	11.55 (2.26)	35.09 (1.81)	22
		Yes	C	11.50 (0.69)	32.56 (0.62)	210
			T	13.96 (2.12)	31.52 (1.61)	25
PPMS	M	No	C	22.26 (2.76)	40.53 (2.56)	19
			T					
		Yes	C	24.41 (2.82)	42.82 (3.27)	17
			T					
	F	No	C	22.64 (2.51)	42.68 (2.46)	22
			T	24.50 (3.81)	33.88^b^ (5.32)	8
		Yes	C	21.24 (1.70)	42.24 (1.84)	34
			T					

*Note.* Comparisons done by linear regression.

a *p* = .088 vs. HLA + /STK11 C. ^b^ *p* = .054 vs. HLA − /STK11 C. ^c^ *p* = .058 vs. HLA + /STK11 C.

### STK11-SNP Reduces Tcell Survival

**Figure 4.**
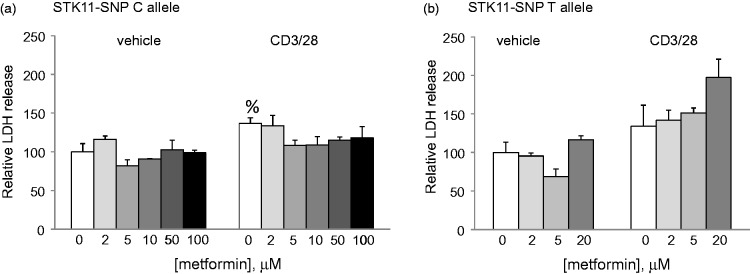
Effect of metformin on PBMC viability. PBMCs were isolated from (a) six MS patients from outside the family who had the major STK11 C allele, or (b) two members of the MSF who have the STK11 T allele. The cells were activated with antibodies to CD3 and CD28 overnight and incubated in the presence of the indicated concentrations of metformin. After 24 hours, cell death was assessed by LDH release into the media. Data are mean ± *SD* of LDH release relative to release in vehicle treated cells in the absence of metformin. ^%^ *p* < .05 vs. vehicle alone.

The STK11 gene encodes LKB1 which by activating AMPK helps maintain cellular ATP levels during increased periods of metabolic stress. When activated, Tcells proliferate and produce large quantities of cytokines which deplete energy reserve. We hypothesized that if the STK11-SNP influenced LKB1 expression, it could affect Tcell viability. To test this, we activated acutely isolated PBMCs with antibodies to CD3 and CD28, in the presence of increasing concentrations of metformin, which induces further oxidative stress by inhibiting mitochondrial function (Figure 4). In cells from MS patients without the STK11-SNP, Tcell activation caused an increase in cell death; however, this was not further increased by up to 100 µM metformin. In contrast, assays carried out using PBMCs from two members of the MSF showed that cell death was increased in CD3 + CD28 activated Tcells at a metformin concentration as low as 20 µM.

## Discussion

Our findings demonstrate that an SNP in intron 5 of the STK11 gene shows a significant association in women with MS and therefore can be considered a genetic risk factor in a similar manner as SNPs identified by GWAS analyses. With *ORs* for females of 1.63 in RRMS, 1.88 in PPMS, and 1.66 for both MS forms, this places the STK11-SNP amongst the highest of reported genetic risk factors identified by GWAS. Although the prevalence of STK11-SNP is 6.8% in the world population, it is only 4% in Caucasian populations, which could explain why it has not been detected in recent GWAS which only included SNPs in Caucasian populations present at 5% or above. That the increase in PPMS women did not reach statistical significance may be due to the smaller group size (56 female PPMS patients compared to 396 female RRMS patients). Alternatively, the development and evolution of PPMS is significantly distinct from that of RRMS patients (Antel et al., 2012), as illustrated herein by the older age of onset. For these reasons, a less significant contribution of the STK11-SNP to the risk of developing PPMS may be due to intrinsic differences between the two types of MS.

In contrast to women, the risk of RRMS was only modestly increased by the STK11-SNP in males (9.1% vs. 8.1%, *OR* = 1.14). Other discordances in SNP prevalence in men versus women have been reported. Polymorphisms in the IFNγ gene can increase or decrease MS risk in men, but not in women (Kantarci et al., 2005), and an SNP in the IL-10 promoter is associated with higher EBV titers in women (Yasui et al., 2008). Gender-dependent differences in these factors could therefore contribute to increased risk of MS in women compared to men.

Identification of the STK11-SNP is based on analyses of the MSF in which a number of women in the maternal lineage had comorbidities of tumors, cancers, cysts, or polyps (TCCP). It was therefore possible that the STK11-SNP is associated with those comorbidities, and not with MS; however, analyses of the reported self-comorbidities as well as of comorbidities in first-degree relatives showed no significant increase in patients harboring the STK11-SNP. We therefore propose that the prevalence of the STK11-SNP in MS patients is not associated with an increase in the presence of cancers or cyst-related comorbidities, but instead to MS disease. In contrast to the MS cohorts, we detected a significant association of STK11-SNP with TCCP comorbidities in the control group, consistent with the known role of LKB1 as a tumor suppressor. While this observation requires further study, it raises the intriguing possibility that the increased inflammatory milieu in MS patients may help restrict the development of cancers and cyst related symptoms, as has been suggested previously (Marrie et al., 2014).

We also tested if the STK11-SNP prevalence was associated with inheritance of HLA-DRB1*1501 allele. In our cohorts, the HLA-DRB1*1501 prevalence was increased about 2.5-fold in MS patients as reported (Schmidt et al., 2007). Stratification by gender showed similar prevalence for men and women in the control group (19.3% vs. 19.7% respectively). and slightly, but not significantly increased prevalence in females versus males MS patients (47.8% vs. 53.9% in RRMS, and 47.2% vs. 53.1% in the PPMS patients, male and female values, respectively). This is in agreement with reports showing a higher prevalence of HLA-DRB1*1501 in women than men (Hensiek et al., 2002; Okuda et al., 2009). Despite slightly higher prevalence in women, the HLA-DRB1*1501 allele was not associated with prevalence of the STK11-SNP, regardless of gender or of type of MS.

The STK11 intron 5 SNP is located 51 bases upstream from the beginning of Exon 6, and it is therefore not obvious how this SNP influences LKB1 expression or function. The majority of mutations in the STK11 gene that are associated with PJS are located within exons leading to missense mutations and truncated proteins (Launonen, 2005). However, several are located in intronic regions (Hastings et al., 2005; Orellana et al., 2013) that can prevent splicing or cause splicing into downstream cryptic splice sites. The STK11-SNP lies upstream of the acceptor splice site for exon 6, suggesting that it does not directly alter splicing events. However, the SNP lies within a canonical half-CRE binding site, suggesting that this region normally can bind CREB, or a related transcription factor, and thereby influence splicing, transcription rate, or initiation site. In agreement with this possibility, several alternate transcripts are predicted for the STK11 gene, and at least two have been experimentally identified. One begins at the same initiation site as the full length LKB1 mRNA, but utilizes an alternative splice site after exon 8 leading to a different C-terminus (Towler et al., 2008). A second alternate transcript has been described (Churchman et al., 1999) which consists of a 444 bp in-frame deletion of exons 5–7 and part of 8. It is therefore possible that transcription factor binding to STK11 intron 5 regulates initiation of downstream mRNAs; if so then the SNP described herein which removes the CRE-half site would be expected to alter those events.

If STK11-SNP is a risk factor for MS, it is important to determine the cell type in which LKB1 deficiency exerts deleterious effects. Two recent papers reported findings using conditional knockout of LKB1 from Schwann cells generated by crossing LKB1 floxed mice with a CNPase-Cre mouse. In both studies, significant hypomyelination of peripheral nerves was observed leading to peripheral neuropathy. In one case, the cause for dysmyelination was attributed to loss of the asymmetric localization of LKB1 (together with the polarity protein Par-3) at the Schwann cell: axon boundary, postulated to be required for correct distribution of myelin components (Shen et al., 2014). In the other, the loss of LKB1 was shown to be associated with reduced mitochondrial oxidative metabolism, causing reductions in citrate, a precursor for acetyl-CoA and lipid synthesis (Pooya et al., 2014). Because the CNPase promoter is expressed in both oligodendrocytes and Schwann cells, it is surprising that CNS myelination appeared normal (Shen et al., 2014). There are several possible explanations for this, for example, cell polarization may not be dependent upon LKB1 in oligodendrocytes, or there may be other sources of acetyl-CoA that can be used by oligodendrocytes for lipid synthesis, for example, from astrocytes which are absent from the PNS. However, the absence of a CNS phenotype in those mice suggests that increased MS risk due to the STK11-SNP is not intrinsic to oligodendrocytes, but instead to another cell type. Whether those are Tcells, parenchymal glial cells, or other remains to be determined.

Whereas the above studies point to a requirement for LKB1 in Schwann cells during early periods of myelination, there is also evidence that LKB1 deficiency in neurons can influence myelination at later time points (Sun et al., 2011). In that study, LKB1 floxed mice were crossed to mice expressing a RIP2-promoter driving Cre-recombinase expression. The RIP2 construct is derived from the rat insulin promoter and drives expression beginning during early development (embryonic day 11.5), primarily in pancreatic β-cells and in a subset of hypothalamic neurons (Gannon et al., 2000). However, this promoter also drives Cre expression in the mid and ventral brain (Wicksteed et al., 2010); and in subset of neurons in the spinal cord (Sun et al., 2010). Those mice developed hind limb paralysis that was accompanied by axonal degeneration in the white matter of the thoracic spinal cord, as well as myelin loss as detected by Luxol fast blue staining. The mice also showed increased inflammation and macrophage infiltration in those regions. Thus, in contrast to deletion of LKB1 from Schwann cells which led to delays and reduced amounts of myelin, deletion of LKB1 from spinal cord neurons resulted in neuronal damage that leads to subsequent demyelination and inflammatory infiltrates. It is therefore possible that expression of the STK11-SNP in neurons also contributes to the observed increased of MS risk.

LKB1 is well characterized as a regulator of cell metabolism under conditions of metabolic stress, and LKB1 deficiency has been shown in several cases to increase cell death. In fibroblasts, LKB1 deficiency was shown to increase cell death following treatment with several metabolic stressors including metformin and H_2_O_2_ (Shaw et al. 2004). In non-small cell lung cancer cells (NSCLCs), treatment with activators of endoplasmic reticulum stress (Inge et al. 2014); or with phenformin (Shackelford et al. 2013), an analog of metformin, selectivity induced apoptosis in the LKB1-deficient NSCLCs. In glioma cells, elevated levels of microRNA-451 decrease LKB1 expression and concomitantly caused an increase in cell death (Godlewski et al. 2010). Most relevant to the current studies, in thymocytes the conditional knockout of LKB1 also reduced survival (Cao et al. 2010). Consistent with these findings, we found that PBMCs isolated from the MSF patients showed increased cell death in the presence of metformin, as compared to PBMCs from MS patients having the major STK11 C allele. One possible explanation is that the presence of the STK11-SNP reduces LKB1 functionality, which reduces the AMPK activation necessary to survive increased metabolic stress. While it is possible that previous medical treatments caused alterations in PBMCs from members of the MSF, neither patient reported having ever received treatment for cysts or fibroids, and neither had been treated for MS symptoms for the previous 9 months before cells were isolated. Nonetheless, further studies are needed to confirm that PBMCs isolated from MS patients with the STK11-SNP show increased sensitivity to metformin independent of any previous medical treatments. While those controls, as well as larger group sizes, are necessary to draw strong conclusions, our findings raise the possibility treatment of MS patients who harbor the STK11-SNP with metformin or other inducer of metabolic stress could be a novel therapeutic approach.

Following activation of PBMCs, increased cellular functions (proliferation and cytokine production) induce a metabolic stress which is normally curbed by the expression and activity of the LKB1 protein, most likely by inducing AMPK activation which in turn shuts down nonrequired cellular processes. This system is efficient, since even further induction of metabolic stress by metformin can be tolerated by cells harboring the major STK11-SNP allele. In contrast, in PBMCs isolated from members of the MSF which have the minor STK11-SNP allele, additional metabolic stress led to cell death.

Our *in vivo* studies suggest that a similar situation may occur in RRMS patients. While neither the presence of the STK11-SNP, nor the HLA-DRB1*1501 allele alone influenced MS severity (as assessed by MSSS), there was significantly reduced severity in female patients harboring both variants. The HLA DR is a membrane bound heterodimeric protein, composed of 2 subunits encoded by the HLA-DRA and HLA-DRB genes, and whose primary function is to present peptide antigens to the Tcell receptors. Variability in the HLA-DRB genes influences antigen binding, and the 1501 allele is thought to increase MS risk by increasing binding to and presentation of aromatic residues present in the myelin basic protein to the Tcell receptor (Ota et al., 1990), leading to increased numbers of MBP-reactive Tcells (Oksenberg et al., 1993). Thus, analogous to the robust Tcell activation induced in PBMCs by antibodies to CD3 and CD28, the HLA-DRB1*1501 could allow for significantly increased Tcell activation. We postulate that when Tcells also harbor the STK11-SNP, they can no longer sustain increased activation, energy supplies are diminished, and cell death ensues. It remains to be reconciled how increased MS risk (due to HLA-DRB1*1501 or to STK11-SNP) can result in eventual lower MS severity as assessed by MSSS. One possibility is that either of these SNPs facilitates triggering of disease by making the immune system more susceptible to activation, leading to increased risk. However, while overall risk is increased, disease severity becomes attenuated in bigenic patients over time, due to loss of activated Tcells (or glial cells), eventually becoming less severe than cohorts who lack the STK11-SNP.

Interactions of the HLA-DRB1*1501 allele with other SNPs which influence MS disease has been previously demonstrated. In combination with an SNP in the CD24 gene, MS patients who were also HLA-DRB1*1501 positive had more severe disease than others (Ghlichnia et al., 2014). Similarly, MS patients who harbored an SNP in the IL2 gene had a higher risk to develop MS, which was further increased in those patients who also had the HLA-DRB1*1501 allele (Sayad, 2014). Combinations that reduce risk have also been reported. An SNP in the ALCAM (activated leukocyte cell adhesion molecule) gene is associated with a higher MS risk and earlier age of onset; however, other SNPs in the ALCAM gene reduce MS risk in HLA-DRB1*1501 individuals (Wagner et al., 2013). Our findings that HLA-DRB1*1501 acts in concert with the STK11 gene are therefore in line with these other observations.

There are several limitations to the current study. One is that only three DNA samples were available from the MSF; therefore, it is not known if the other two siblings have the same STK11-SNP, or if one of the parents is homozygous for this SNP (presumably the mother who has symptoms consistent with PJS). However, there are several reasons to believe that the three members seen have MS. First, most recent neurological examinations assigned MSF1 an EDSS of 1; and MSF4 an EDSS of 6, although MSF2 (the identical twin of MSF1) had an EDSS of 0. Second, medical records of MSF1, MSF2, and MSF4 show history of relapses and clinical episodes since initial diagnoses in 2006 and 2007. Third, recent MRI of MSF4 revealed lesions typical of MS, including periventricular lesions emanating from the third ventricle (Dawson fingers). Fourth, IgG oligoclonal bands were found in CSF of MSF1; and MSF4 had a high IgG index but no oligoclonal bands. Although MSF2 has been diagnosed with CIS, conversion to MS only occurs in about two thirds of the patients (Brownlee and Miller, 2014). It is possible that the other members of the MSF have phenotypes similar but distinct from MS, such as Pelizaeus-Merzbacher disease; in which case the presence of the STK11-SNP may be serendipitous, or may be a common risk factor for a number of illnesses. Another limitation is the relatively small size of our PPMS group (100 total patients), which may account for the lack of statistical significance with respect to STK11-SNP being a predictor for MS, or its association with the HLA-DRB1*1501 allele. A third limitation is that our *in vitro* studies were limited to two samples from the MSF; we may find that upon further testing (as cells become available), that increased sensitivity to metformin is not associated with the STK11-SNP but to some other factor shared by members of this family. However, multiple reports that LKB1 deficiency increases sensitivity to metabolic stress suggest that a similar phenomenon could occur in these PBMCs.

In summary, our findings indicate that STK11 intron 5 SNP is increased in a large cohort of RRMS and PPMS patients, with a significantly higher association with MS in women. Studies to identify other SNPs inherited together with STK11-SNP, and that could act in concert to increase MS risk are ongoing. Our *in vitro* findings, while limited, allow us to generate hypotheses that could help to explain the reduced disease severity in MS patients who have the STK11-SNP and the HLA-DRB*1501 allele. It is possible that LKB1 deficiency (due to the STK11-SNP or to other variants that influences LKB1 activity) sensitizes cells to increased metabolic stress, leading to gradual loss of highly activated Tcells, and eventually a reduction in MSSS. Because many MS patients are prescribed metformin to treat type 2 diabetes, a retrospective analysis could provide insight as to whether metformin has any beneficial effect on MS disease severity.
